# HRES-1/Rab4 Promotes the Formation of LC3^+^ Autophagosomes and the Accumulation of Mitochondria during Autophagy

**DOI:** 10.1371/journal.pone.0084392

**Published:** 2014-01-03

**Authors:** Gergely Talaber, Gabriella Miklossy, Zachary Oaks, Yuxin Liu, Sharon A. Tooze, Dmitriy M. Chudakov, Katalin Banki, Andras Perl

**Affiliations:** 1 Departments of Medicine, State University of New York, Upstate Medical University, Syracuse, New York, United States of America; 2 Biochemistry and Molecular Biology, State University of New York, Upstate Medical University, Syracuse, New York, United States of America; 3 Microbiology and Immunology, State University of New York, Upstate Medical University, Syracuse, New York, United States of America; 4 Cancer Research UK London Research Institute, London, England, United Kingdom; 5 Shemiakin-Ovchinnikov Institute of Bioorganic Chemistry, RAS, Moscow, Russia; 6 Department of Pathology, State University of New York, Upstate Medical University, Syracuse, New York, United States of America; University of Michigan, United States of America

## Abstract

HRES-1/Rab4 is a small GTPase that regulates endocytic recycling. It has been colocalized to mitochondria and the mechanistic target of rapamycin (mTOR), a suppressor of autophagy. Since the autophagosomal membrane component microtubule-associated protein light chain 3 (LC3) is derived from mitochondria, we investigated the impact of HRES-1/Rab4 on the formation of LC3^+^ autophagosomes, their colocalization with HRES-1/Rab4 and mitochondria, and the retention of mitochondria during autophagy induced by starvation and rapamycin. HRES-1/Rab4 exhibited minimal baseline colocalization with LC3, which was enhanced 22-fold upon starvation or 6-fold upon rapamycin treatment. Colocalization of HRES-1/Rab4 with mitochondria was increased >2-fold by starvation or rapamycin. HRES-1/Rab4 overexpression promoted the colocalization of mitochondria with LC3 upon starvation or rapamycin treatment. A dominant-negative mutant, HRES-1/Rab4^S27N^ had reduced colocalization with LC3 and mitochondria upon starvation but not rapamycin treatment. A constitutively active mutant, HRES-1/Rab4^Q72L^ showed diminished colocalization with LC3 but promoted the partitioning of mitochondria with LC3 upon starvation or rapamycin treatment. Phosphorylation-resistant mutant HRES-1/Rab4^S204Q^ showed diminished colocalization with LC3 but increased partitioning to mitochondria. A newly discovered C-terminally truncated native isoform, HRES-1/Rab4^1–121^, showed enhanced localization to LC3 and mitochondria without starvation or rapamycin treatment. HRES-1/Rab4^1–121^ increased the formation of LC3^+^ autophagosomes in resting cells, while other isoforms promoted autophagosome formation upon starvation. HRES-1/Rab4, HRES-1/Rab4^1–121^, HRES-1/Rab4^Q72L^ and HRES-1/Rab4^S204Q^ promoted the accumulation of mitochondria during starvation. The specificity of HRES-1/Rab4-mediated mitochondrial accumulation is indicated by its abrogation by dominant-negative HRES-1/Rab4^S27N^ mutation. The formation of interconnected mitochondrial tubular networks was markedly enhanced by HRES-1/Rab4^Q72L^ upon starvation, which may contribute to the retention of mitochondria during autophagy. The present study thus indicates that HRES-1/Rab4 regulates autophagy through promoting the formation of LC3^+^ autophagosomes and the preservation of mitochondria.

## Introduction

Autophagy is a complex stress-induced catabolic process that breaks down and recycles organelles and cytoplasmic materials [1]. While autophagy is a fundamental mechanism of cell survival, its dysregulation has been widely implicated in cancer, inflammatory, and neurodegenerative diseases [1], [2]. During autophagy, a new organelle, the autophagosome, is assembled from membrane components of mitochondria [3], plasma membrane [4] and endoplasmic reticulum [5]. Recycling endosomes have been suggested to contribute to the biogenesis of autophagosomes [6]. A signature component of the autophagosome membrane is microtubule-associated protein light chain 3 (LC3) [7], which has been widely identified as a binding partner of endosomal traffic regulator Rab GTPases [8], [9]. The early endosomal marker Rab11 has been implicated in autophagosome formation [6], while the late endosomal Rab7 contributes to endosome maturation [9] and progression [10].

We have previously documented that the small GTPase HRES-1/Rab4 regulated the recycling and lysosomal degradation of surface receptors, GLUT4 on adipocytes [11] as well as CD4 [12] and CD3ζ in T lymphocytes [13]. Expression of HRES-1/Rab4 is redox-controlled: induced by H_2_O_2_ and inhibited by glutathione [13]. HRES-1/Rab4 was colocalized with the lysosomes and the mechanistic target of rapamycin (mTOR), which acts as a suppressor of autophagy [14], [15]. Blockade of mTOR with rapamycin, which is an effective treatment in patients with systemic lupus erythematosus [16], [17], inhibited the oxidative stress-induced expression of HRES-1/Rab4 and the lysosomal degradation of CD4 and CD3ζ [13]. Along these lines, mTOR has been localized to endosomes [18], including those carrying HRES-1/Rab4 [13]. Overexpression of HRES-1/Rab4 and activation of mTOR can be detected in T cells of patients with systemic lupus erythematosus (SLE) [13] and lupus-prone mice, where autophagy appears to be involved in disease pathogenesis [19]. While HRES-1/Rab4 promotes the lysosomal degradation of proteins by autophagy, it appears to inhibit the autophagy of mitochondria or mitophagy [19]. The biogenesis of LC3^+^ autophagosomes is dependent on the supply of membranes from mitochondria [3]. In order to test the hypothesis that HRES-1/Rab4 influences the formation of autophagosomes, we investigated the colocalization of its wild-type and functionally distinct mutant isoforms with LC3 and mitochondria during autophagy, which was induced by starvation or mTOR blockade with rapamycin in HeLa cells, owing to the limited size and paucity of organelles in primary human T lymphocytes. Here, we show that HRES-1/Rab4 colocalizes with the autophagosomal membrane component LC3 and promotes its partitioning to the mitochondria during autophagy. Dominant-negative HRES-1/Rab4^S27N^ mutation [20] blocked colocalization with LC3 and partitioning to the mitochondria induced by starvation but not by rapamycin. Constitutively active HRES-1/Rab4^Q72L^ [21] showed diminished colocalization with LC3 but promoted partitioning of mitochondria with LC3 upon starvation or rapamycin treatment. A newly discovered C-terminally truncated natural isoform, HRES-1/Rab4^1–121^, showed enhanced localization to LC3 and its partitioning to mitochondria without starvation or rapamycin treatment. CDC kinase phosphorylation-deficient HRES-1/Rab4^S204Q^ [22] showed diminished colocalization with LC3 but facilitated its partitioning to mitochondria without starvation or rapamycin treatment. Only HRES-1/Rab4^1–121^ increased the formation of LC3^+^ autophagosomes in resting cells, while other isoforms promoted autophagosome formation upon starvation. HRES-1/Rab4, HRES-1/Rab4^1–121^, HRES-1/Rab4^Q72L^ and HRES-1/Rab4^S204Q^, but not HRES-1/Rab4^S27N^, enhanced the accumulation of mitochondria during starvation. The formation of interconnected mitochondrial tubular networks was enhanced by HRES-1/Rab4^Q72L^ upon starvation, which may contribute to the preservation of mitochondria during autophagy. Overall, these results reveal a new role of HRES-1/Rab4 in autophagy and mitochondrial homeostasis.

## Results

### HRES-1/Rab4 colocalizes with the autophagosomal membrane component LC3

**Figure 1:**
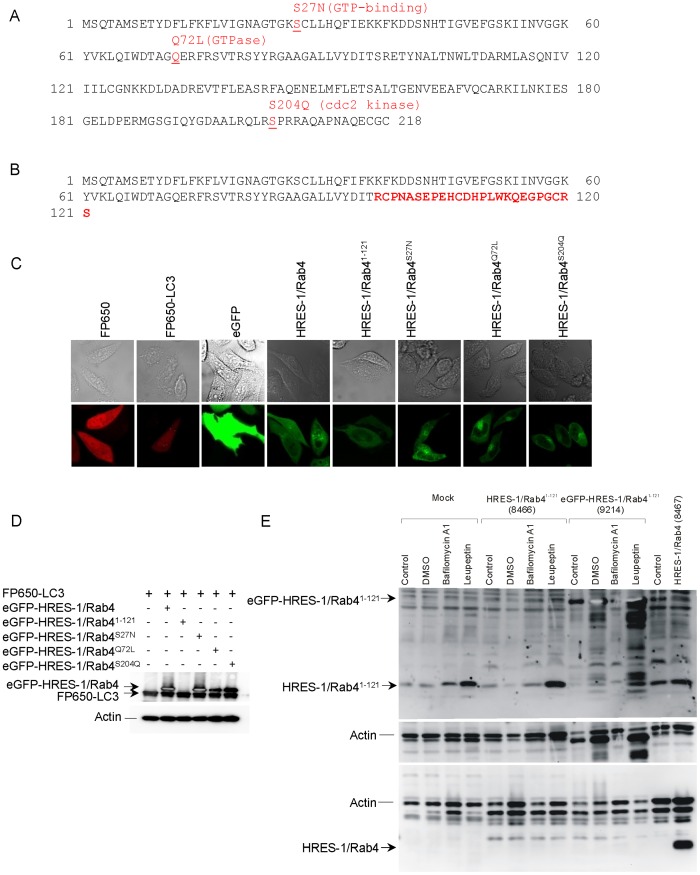
Detection of LC3 fused to FP650 (FP650-LC3) and HRES-1/Rab4 isoforms, including wild-type HRES-1/Rab4, C-terminally truncated HRES-1/Rab4^1–121^, dominant-negative/GTP binding-deficient HRES-1/Rab4^S27N^, constitutively active/GTPase-deficient HRES-1/Rab4^Q72L^ and phosphorylation-resistant form HRES-1/Rab4^S204Q^, tagged with eGFP. A, Functional domains of proteins encoded by the HRES-1/Rab4 cDNA at 1q42 [12] (Genbank accession number: AY585832). Amino acid changes previously shown to affect Rab4 activity are typed in red. HRES-1/Rab4^S27N^ prevents GTP binding and acts as a dominant negative mutation [20]. HRES-1/Rab4^Q72L^ is constitutively active due to elimination of GTPase activity [21]. HRES-1/Rab4^S204Q^ will not be phosphorylated by p34cdc2 kinase in mitotic cells and remains endosome-associated throughout the cell cycle [22]. B, Amino acid sequence of HRES-1/Rab4^1–121^, representing a 36-nucleotide out-of-frame deletion, is attributed to alternative splicing (GenBank submission number 1591873). This results in a frameshift with an amino acid sequence corresponding to the 96 N-terminal residues of HRES-1/Rab4 continuing into 25 C-terminal residues (typed in red characters), which are unrelated to the amino acid sequence of residues 97-218 in wild-type HRES-1/Rab4. C, Confocal microscopy of HeLa cells transfected with expression vectors producing FP650-LC3 (emitting red fluorescence) and HRES-1/Rab4 isoforms fused to eGFP (emitting green fluorescence) relative to control cells transfected with vectors expressing fluorescent proteins FP650 and eGFP alone. D, Western blot analysis of HeLa cells transduced with expression vectors producing eGFP-HRES-1/Rab4 and FP650-LC3 fusion proteins. HRES-1/Rab4 isoforms were detected with antibody SC312 directed to the C-terminus which is absent in HRES-1/Rab4^1–121^. E, Western blot analysis of HRES-1/Rab4^1–121^ expression in HeLa cells transfected with pAAV-HRES-1/Rab4^1–121^-IRES-GFP vector (clone 8466), pAAV-hrGFP-HRES-1/Rab4^1–121^ vector (clone 9214), and pAAV-HRES-1/Rab4-IRES-GFP vector (clone 8467). Cells were incubated without (control) or with 0.1% DMSO, bafilomycin A1 (200 nM), or leupeptin (10 μg/ml). HRES-1/Rab4^1–121^ and HRES-1/Rab4^1–121^-GFP fusion protein were detected with rabbit antibody G1432. HRES-1/Rab4 was detected with rabbit antibody 13407 [12].

The colocalization of HRES-1/Rab4 and LC3 was investigated in HeLa cells transfected with LC3 fused to FP650 (FP650-LC3) and HRES-1/Rab4 isoforms, including wild-type HRES-1/Rab4, HRES-1/Rab4^S27N^, HRES-1/Rab4^Q72L^, HRES-1/Rab4^S204Q^, and C-terminally truncated HRES-1/Rab4^1–121^, each tagged to the C-terminus of eGFP (Fig. 1). Among the mutant isoforms, HRES-1/Rab4^S27N^ acts as a dominant negative mutation that prevents GTP binding [20]. HRES-1/Rab4^Q72L^ is constitutively active due to elimination of GTPase activity [21]. HRES-1/Rab4^S204Q^ cannot be phosphorylated by p34cdc2 kinase in mitotic cells and remains endosome-associated throughout the cell cycle [22]. HRES-1/Rab4^1–121^ is encoded by a newly identified alternatively spliced mRNA that represents a 36-nucleotide out-of-frame deletion (GenBank submission number: 1591873). This results in a frame shift with an amino acid sequence corresponding to the 96 N-terminal residues of HRES-1/Rab4 continuing into 25 C-terminal residues unrelated to residues 97–218 of wild-type HRES-1/Rab4 (Fig. 1B).

**Figure 2:**
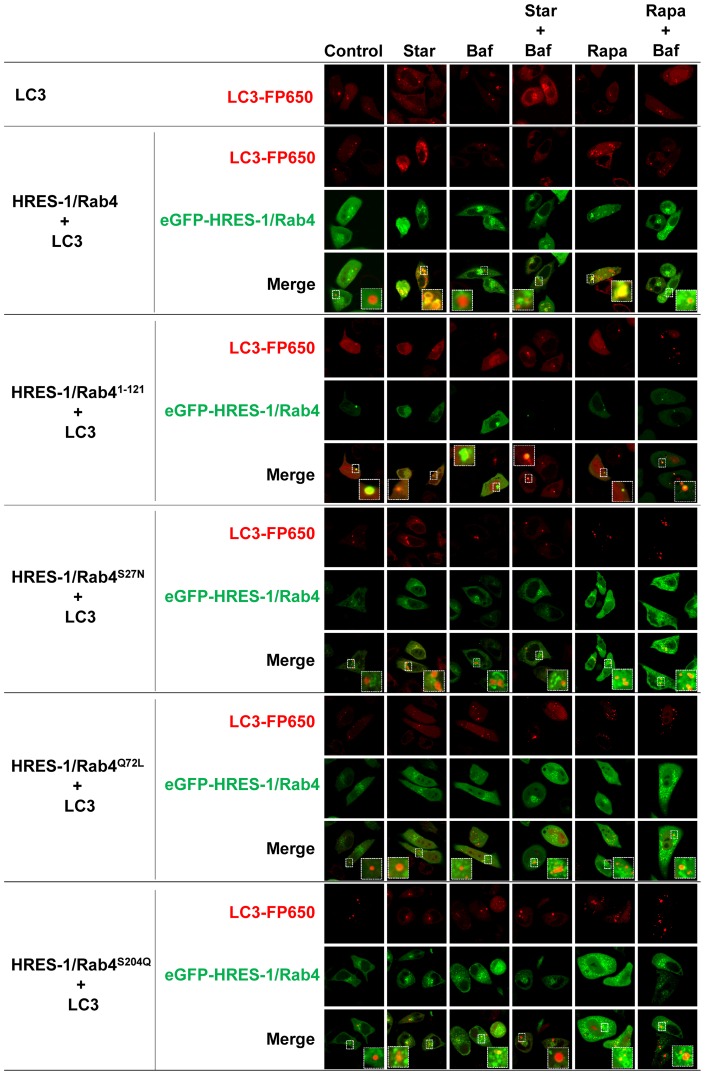
Confocal microscopy of HeLa cells transfected with expression vector producing LC3 tagged with FP650 (emitting red fluorescence) and different isoforms of HRES-1/Rab4, including wild-type (HRES-1/Rab4), C-terminally truncated form (HRES-1/Rab4^1–121^), dominant-negative form (Rab4^S27N^), constitutively active (HRES-1/Rab4^Q72L^) and phosphorylation-resistant form (HRES-1/Rab4^S204Q^) tagged with eGFP (emitting green fluorescence). Cells were kept in complete medium (Control), starved in serum-free medium without glutamine (Star), or treated with autophagy-modifying agents, bafilomycin A1 (Baf) and rapamycin (Rapa) for 4 hours. Starved and rapamycin treated cells were also treated with Baf. Images are representative of 6–40 cells analyzed during three independent experiments. Image inserts bracketed by broken lines correspond to LC3 vesicles magnified from the original image. Individual and composite color channels are shown for each experimental condition.

**Figure 3:**
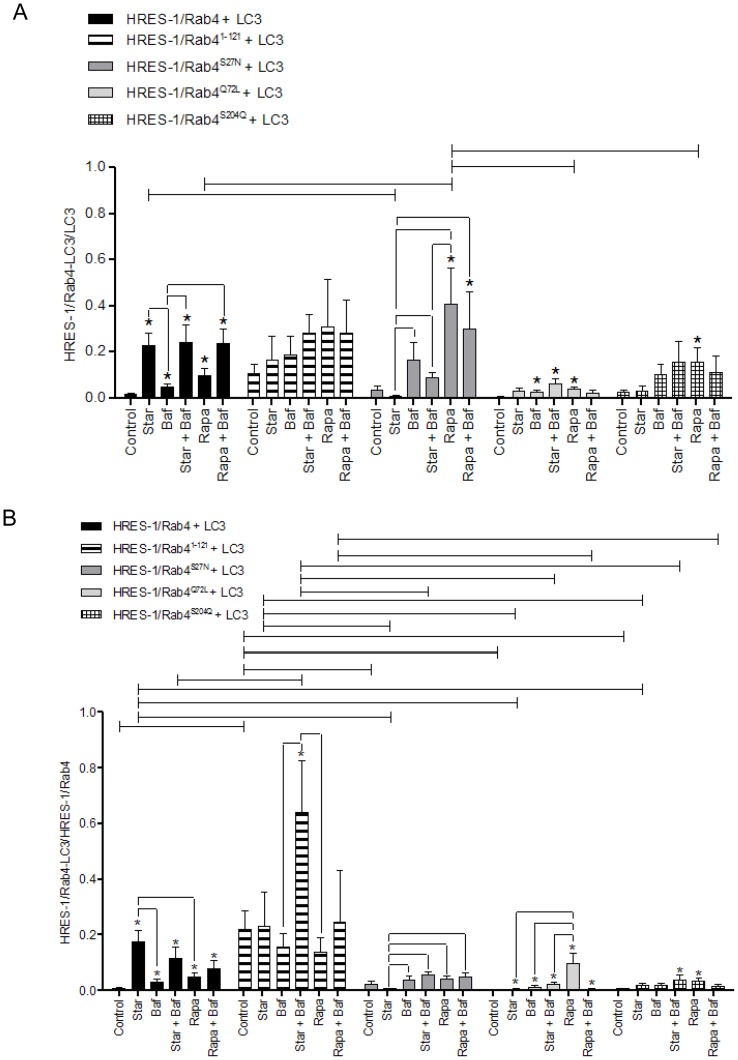
Cumulative analysis of colocalization of HRES-1/Rab4 and LC3 induced by starvation and rapamycin. Colocalization of HRES-1/Rab4 and LC3 was quantified relative to total LC3 (panel A) and total HRES-1/Rab4 content (panel B). The colocalization of HRES-1/Rab4 with LC3 was most profoundly skewed by HRES-1/Rab4^S27N^ which blocked colocalization under starvation and promoted colocalization during mTOR blockade. In contrast to HRES-1/Rab4^S27N^, HRES-1/Rab4^Q72L^ blocked colocalization with LC3 both under starvation and mTOR blockade. Data represent mean ± SEM of 6–29 cells acquired in 3 independent experiments. * indicates p values<0.05 reflecting comparison to control cells among cell cultures transfected with the same construct using paired two-tailed t-tests; brackets connecting bars within each construct also reflect comparison with paired two-tailed t-tests. Brackets connecting bars between constructs reflect p<0.05 using ANOVA followed by Bonferroni's post-test.

Expression of transduced eGFP-HRES-1/Rab4 and FP650-LC3 fusion proteins was monitored by fluorescence microscopy (Figs. 1C), flow cytometry (Figs. S1A and B), and western blot (Figs. 1D and E). The colocalization of HRES-1/Rab4 isoforms and LC3 was analyzed by confocal microscopy (Fig. 2) and quantified relative to both the total LC3 (Fig. 3A) and total HRES-1/Rab4 signal pools (Fig. 3B). HRES-1/Rab4 formed round-shaped structures around LC3^+^ vesicles. (Fig. 2). While minimal colocalization was observed between HRES-1/Rab4 and LC3 in cells cultured in complete medium (1.6±0.5%; Fig. 3), starvation initiated by withdrawal of serum and glutamine for 4 h enhanced their colocalization 22-fold (23.0±5.4%, p = 0.003; Figs. 3A). Colocalization was also induced, however, to a lesser extent, 6-fold, after exposure to rapamycin (Rapa) (9.8±3.5%; p = 0.020; Fig. 3A). Unlike starvation-induced colocalization, the effect of Rapa was not reduced by balifomycin A1 (Baf). HRES-1/Rab4^1–121^ showed the most robust colocalization with LC3 but no responsiveness to starvation or Rapa. The disproportionately strong colocalization of HRES-1/Rab4^1–121^ with LC3 may be related to an instability and susceptibility to lysosomal degradation of this truncated isoform (Fig. 1E). Colocalization of HRES-1/Rab4^S27N^ with LC3 was induced 11.6-fold (p = 0.020) by Rapa but not starvation (Fig. 3A). Colocalization of HRES-1/Rab4^Q72L^ with LC3 was robustly induced by Rapa (70-fold; p = 0.0005) and reversed by Baf (p = 0.04; Fig. 3A). HRES-1/Rab4^S204Q^ was least prone to colocalize with LC3 upon starvation (Fig. 3A). The resistance of HRES-1/Rab4^S27N^, HRES-1/Rab4^Q72L^, and HRES-1/Rab4^S204Q^ to starvation-induced colocalization with LC3 implies that this process is dependent on GTP binding, GTPase activity, and phosphorylation at S204.

The fraction of LC3 colocalizing with the HRES-1/Rab4 was increased to 0.23±0.06 upon starvation from 0.015±0.006 (p = 0.0001) in complete medium (Fig. 3A). While Baf alone moderately increased this ratio (0.046±0.015, p = 0.02) relative to cells grown in complete medium, it failed to influence the impact of starvation. The ratio of LC3 bound to HRES-1/Rab4 was increased by Rapa to 0.098±0.03 (p = 0.001) and further augmented by the combination of Rapa and Baf to 0.24±0.06 (p = 0.00005). This pattern of changes was influenced by using mutated HRES-1/Rab4 isoforms. LC3 showed the greatest colocalization with C-terminally truncated HRES-1/Rab4^1–121^ at baseline and the least responsiveness to starvation or treatment with Rapa or Baf, suggesting that this naturally occurring HRES-1/Rab4 isoform has limited functional capacity. LC3 failed to colocalize with HRES-1/Rab4^S27N^ in complete medium or upon starvation. However, Rapa markedly increased the ratio of HRES-1/Rab4-positive LC3 vesicles to 0.41±0.16 from 0.035±0.018 at baseline (p = 0.02). Baf did not influence the colocalization of LC3 with HRES-1/Rab4^S27N^ in the absence or presence of Rapa. The colocalization of LC3 with HRES-1/Rab4^Q72L^ and LC3 was moderately responsive to Baf, starvation combined with Baf, and Rapa. The Rapa-induced colocalization of LC3 with HRES-1/Rab4^Q72L^ was reduced at 0.037±0.01 relative to that with HRES-1/Rab4^S27N^ at 0.41±0.16 (p = 0.029; Fig. 3A). Starvation did not influence to the colocalization of LC3 with HRES-1/Rab4^Q72L^. LC3 also showed increased colocalization with HRES-1/Rab4^S204Q^ upon treatment with Rapa (p = 0.005). 2-way ANOVA and Bonferroni post-tests revealed resistance to starvation-induced colocalization of LC3 to mutated HRES-1/Rab4 isoforms and greatest colocalization to HRES-1/Rab4^S27N^ in response to treatment with Rapa (Fig. 3A).

We also quantified the ratio of HRES-1/Rab4 colocalizing with LC3 relative to total LC3 (Fig. 3A) or HRES-1/Rab4 (Fig. 3B). Starvation induced the fraction of HRES-1/Rab4 colocalizing with LC3 from 0.009±0.003 in complete medium to 0.17±0.04 upon starvation (p = 0.00007). The ratio of HRES-1/Rab4 colocalized with LC3 was also increased by Rapa at 0.05±0.02 (p = 0.006), but this was less pronounced than the impact of starvation (p = 0.015). HRES-1/Rab4^1–121^ was most prominently associated with LC3 at baseline 0.22±0.06 relative to any other isoform tested (p<0.01), and that was further augmented by starvation and Baf to 0.64±0.18 (p = 0.03, Fig. 2B). HRES-1/Rab4^S27N^ was resistant to starvation-induced colocalization with LC3 relative to wild-type HRES-1/Rab4 (ANOVA p<0.01; Fig. 3B). HRES-1/Rab4^Q72L^ failed to colocalize with LC3 upon starvation, but it was effectively induced by Rapa to 0.096±0.0036 from a baseline of 0.0013±0.0006 (p = 0.0055) in complete medium (Fig. 3B). Colocalization of HRES-1/Rab4^S204Q^ with LC3 was also induced by Rapa at 0.034±0.01 (p = 0.005, Fig. 3B). 2-way ANOVA and Bonferroni post-tests revealed an increased localization of HRES-1/Rab4^1–121^ to LC3 over all other HRES-1/Rab4 isoforms. Starvation-induced colocalization of HRES-1/Rab4 with LC3 was blocked by C-terminal truncation in HRES-1/Rab4^1–121^, inability to bind GTP, to exert GTPase activity, or to respond to phosphorylation at S204 (Fig. 3B).

### Colocalization of LC3 with mitochondria is modulated by HRES-1/Rab4

**Figure 4:**
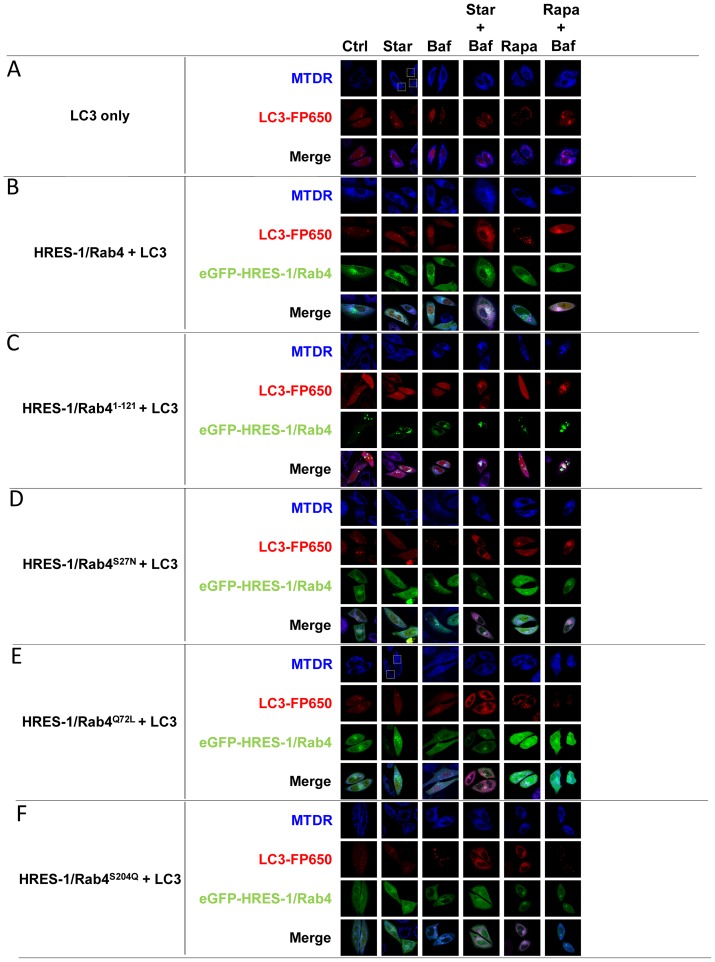
Confocal microscopy of HRES-1/Rab4, mitochondria, and LC3^+^ autophagosomes in HeLa cells under starvation (Star) and treatment with rapamycin (Rapa) and bafilomycin A1 (Baf). eGFP-tagged HRES-1/Rab4 isoforms were identified by green fluorescence. Mitochondria were stained with MTDR and visualized by blue fluorescence. LC3^+^ autophagosomes were visualized by red fluorescence of FP650-LC3. Individual and composite color channels are shown for each experimental condition. A, HeLa cells were transfected with FP650-LC3 alone. B, HeLa cells were transfected with FP650-LC3 and eGFP-tagged HRES-1/Rab4. C, HeLa cells were transfected with FP650-LC3 and eGFP-tagged HRES-1/Rab4^1–121^. D, HeLa cells were transfected with FP650-LC3 and eGFP-tagged HRES-1/Rab4^S27N^. E, HeLa cells were transfected with FP650-LC3 and eGFP-tagged HRES-1/Rab4^Q72L^. F, HeLa cells were transfected with FP650-LC3 and eGFP-tagged HRES-1/Rab4^S204Q^. The areas showing the formation of mitochondrial tubular networks are delineated by white dotted rectangles in panels A and E.

**Figure 5:**
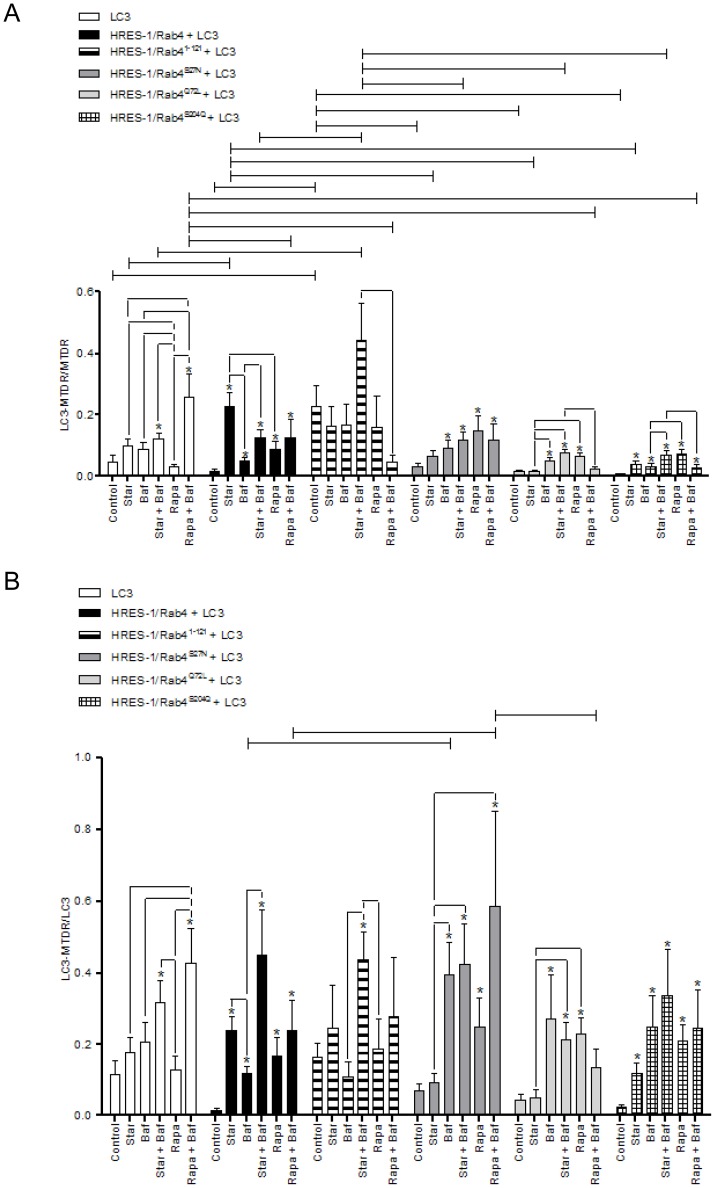
Quantitative analysis of colocalization between LC3^+^ autophagosomes and mitochondria in HeLa cells transfected with eGFP-tagged HRES-1/Rab4 isoforms and FP650-LC3. Autophagy was induced by starvation (Star) or treatment with rapamycin (Rapa) in the presence or absence of bafilomycin A1 (Baf). A, Colocalization of FP-650 LC3 and MTDR-stained mitochondria relative to the total mitochondrial pool. B, Colocalization of FP-650 LC3 and MTDR-stained mitochondria relative to the total LC3 pool. Data represent mean ± SEM of 6–30 cells acquired in 3 independent experiments. * indicates p values<0.05 reflecting comparison to control cells among cell cultures transfected with the same construct using paired two-tailed t-tests; brackets connecting bars within each construct also reflect comparison with paired two-tailed t-tests. Brackets connecting bars between constructs reflect p<0.05 using ANOVA followed by Bonferroni's post-test.

After we found significant colocalization of HRES-1/Rab4 with LC3 upon starvation, we examined whether the colocalization between LC3 and MTDR, was affected by HRES-1/Rab4. As evident from representative images (Fig. 4) and cumulative analyses (Figs. 5), there was limited MTDR-LC3 colocalization at baseline, except with introduction of HRES-1/Rab4^1–121^. Upon starvation, wild-type HRES-1/Rab4 increased the ratio of LC3^+^ mitochondria/total mitochondria to 0.226±0.049 from 0.015±0.005 at baseline (p = 4.8×10^−5^) and from 0.096±0.024 with starvation in the presence of transduced LC3 alone (p = 0.0229, Fig. 5A). In contrast, mutated HRES-1/Rab4^1–121^, HRES-1/Rab4^S27N^, HRES-1/Rab4^Q72L^, and HRES-1/Rab4^S204Q^ limited the formation of LC3^+^ mitochondria upon starvation (Figs. 4 and 5A). HRES-1/Rab4 also promoted the Rapa-induced formation of LC3^+^ mitochondria (0.087±0.027; p = 0.0008 relative to baseline), which was less pronounced with HRES-1/Rab4^Q72L^ and HRES-1/Rab4^S204Q^. In the presence of Rapa and Baf, the latter being a lysosomal inhibitor, the fraction of LC3^+^ mitochondria was markedly enhanced to 0.259±0.111 relative to untreated controls (0.046±0.022; p = 0.0008, Fig. 5A). This phenomenon, i.e. Rapa-induced colocalization of mitochondria with LC3 in the presence of Baf, was obliterated by HRES-1/Rab4^Q72L^ and HRES-1/Rab4^S204Q^ (Fig. 5A).

In contrast to the strong influence of HRES-1/Rab4 on partitioning of mitochondria to LC3 (Fig. 5A), the influence on partitioning of LC3 to mitochondria was modest (Fig. 5B). Interestingly, the partitioning of LC3 to mitochondria was reduced from 0.112±0.041 to 0.011±0.004 in the presence of HRES-1/Rab4 at baseline (p = 0.042; Fig. 5B). During starvation, HRES-1/Rab4 and HRES-1/Rab4^S204Q^ promoted the partitioning of LC3 to mitochondria at 0.239±0.042 (p = 2.9×10^−7^) and 0.115±0.031 (p = 0.004), respectively (Fig. 5B). The greatest shift of LC3 to mitochondria was elicited by HRES-1/Rab4^S27N^ in the presence of Rapa and Baf (0.586±0.263) relative to baseline (0.067±0.022; p = 0.010; Fig. 5B). The Rapa/Baf-induced partitioning of LC3 to mitochondria was diminished by wild-type HRES-1/Rab4 and HRES-1/Rab4^Q72L^ (Fig. 5B).

### Starvation and bafilomycin A1 (Baf) induce colocalization of HRES-1/Rab4 with mitochondria

**Figure 6:**
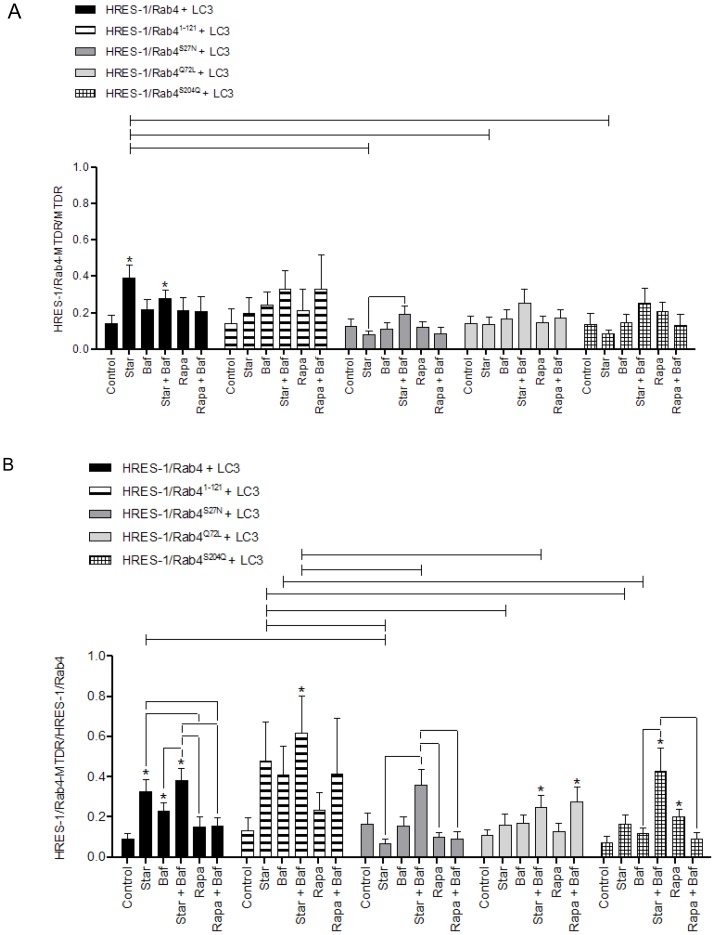
Quantitative analysis of colocalization between HRES-1/Rab4 and mitochondria in HeLa cells transfected with eGFP-tagged HRES-1/Rab4 isoforms and FP650-LC3. Autophagy was induced by starvation (Star) or treatment with rapamycin (Rapa) in the presence or absence of bafilomycin A1 (Baf). A, Colocalization of HRES-1/Rab4 and MTDR-stained mitochondria relative to the total mitochondrial pool. B, Colocalization of HRES-1/Rab4 and MTDR-stained mitochondria relative to the total HRES-1/Rab4 pool. Bars represent mean ± SEM. Data represent mean ± SEM of 6–29 cells acquired in 3 independent experiments. * indicates p values<0.05 reflecting comparison to control cells among cell cultures transfected with the same construct using paired two-tailed t-tests; brackets connecting bars within each construct also reflect comparison with paired two-tailed t-tests. Brackets connecting bars between constructs reflect p<0.05 using ANOVA followed by Bonferroni's post-test.

During autophagy, proteins and organelles alike are carried to the lysosome as endosomal cargo [23].Since HRES-1/Rab4 influenced the colocalization of mitochondria with LC3, we investigated if mitochondria were associated with HRES-1/Rab4-carrying endosomes. As shown in Fig. 4B, HRES-1/Rab4 colocalized with mitochondria. Starvation strongly promoted the association of HRES-1/Rab4 with mitochondria (Fig. 6). HRES-1/Rab4^1–121^ exhibited the greatest colocalization with mitochondria, approaching 65% in response to starvation and Baf (Fig. 6). HRES-1/Rab4^Q72L^ also showed colocalization with mitochondria in response to starvation and Baf or Rapa and Baf (Fig. 6).Starvation did not induce the partitioning of HRES-1/Rab4^S27N^ or HRES-1/Rab4^S204Q^ to mitochondria. Rapa only induced the colocalization of HRES-1/Rab4^S204Q^ to mitochondria, which was reversed by Baf (Fig. 6). Thus, Baf promoted the association of HRES-1/Rab4^Q72L^ but inhibited the association of HRES-1/Rab4^S204Q^ with mitochondria in the presence of Rapa (Fig. 6).

### Effect of HRES-1/Rab4 on the formation of LC3^+^ autophagosomes

**Figure 7:**
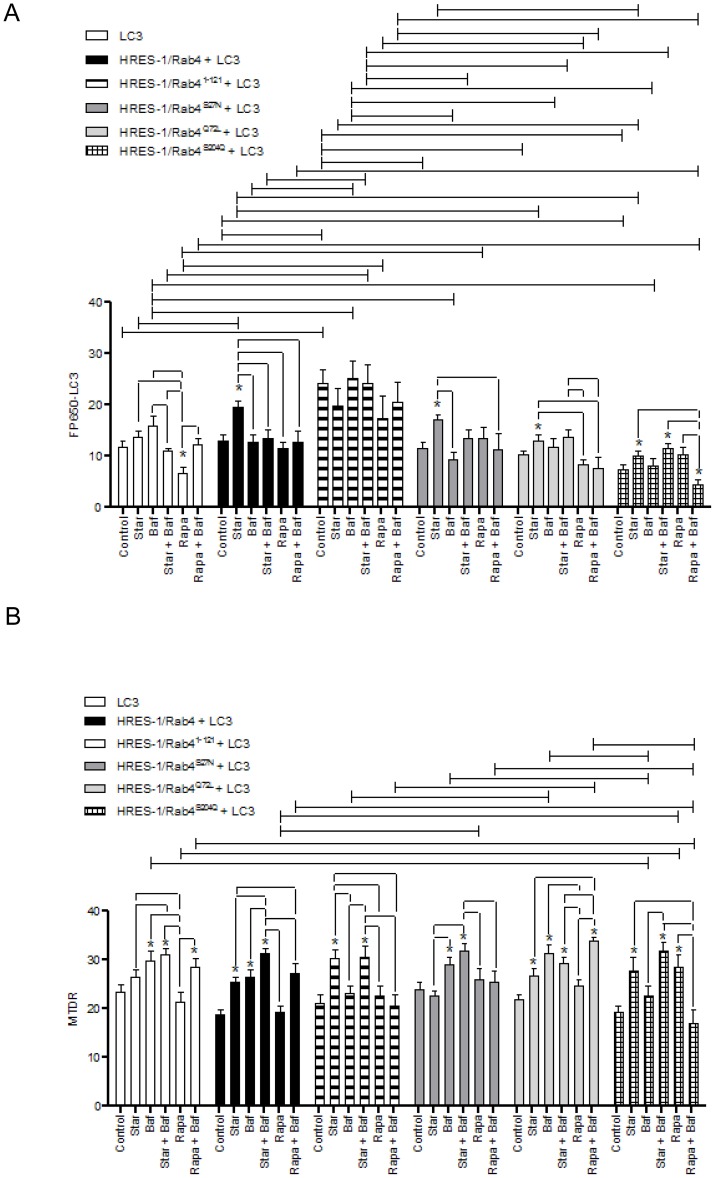
Quantitative analyses of the effect by HRES-1/Rab4 on the accumulation of LC3^+^ autophagosomes (panel A) and MTDR-stained mitochondria (panel B). HeLa cells were transfected with eGFP-tagged HRES-1/Rab4 isoforms and FP650-LC3. Autophagy was induced by starvation (Star) or treatment with rapamycin (Rapa) in the presence or absence of bafilomycin A1 (Baf). Data represent mean ± SEM of 6–30 cells acquired in 3 independent experiments. * indicates p values<0.05 reflecting comparison to cells transfected with LC3 alone using paired two-tailed t-tests; brackets connecting bars within each construct also reflect comparison with paired two-tailed t-tests. Brackets connecting bars between constructs reflect p<0.05 using ANOVA followed by Bonferroni's post-test.

Among the HRES-1/Rab4 isoforms, only HRES-1/Rab4^1–121^ increased the formation of LC3^+^ autophagosomes in the absence of starvation or Rapa treatment, using two-way ANOVA and Bonferroni's post-test comparison (Fig. 7A). Under starvation, autophagosome formation was enhanced by wild-type HRES-1/Rab4, HRES-1/Rab4^S27N^, HRES-1/Rab4^Q72L^, and HRES-1/Rab4^S27N^, using paired t-test (Fig. 7A). Relative to starvation, Rapa reduced the formation of LC3^+^ autophagosomes with overexpression of HRES-1/Rab4 and HRES-1/Rab4^Q72L^. This effect of Rapa was sustained in the presence of Baf (Fig. 7A).

### HRES-1/Rab4, HRES-1/Rab4^1–121^, HRES-1/Rab4^Q72L^ and HRES-1/Rab4^S204Q^ promote the accumulation of mitochondria during starvation

We investigated the impact of HRES-1/Rab4 on the mitochondrial mass of HeLa cells. As opposed to primary lymphocytes and Jurkat T cells [19], HRES-1/Rab4 did not influence the accumulation of mitochondria in resting HeLa cells. However, HRES-1/Rab4, HRES-1/Rab4^1–121^, HRES-1/Rab4^Q72L^ and HRES-1/Rab4^S204Q^ promoted the accumulation of mitochondria during starvation (Fig. 7B). In contrast, dominant-negative HRES-1/Rab4^S27N^ did not have such effect (Fig. 7B). Rapa only promoted the accumulation of mitochondria in the presence of HRES-1/Rab4^S204Q^, which was reversed by Baf (Fig. 7B). In contrast, Rapa promoted the accumulation of mitochondria in the presence of HRES-1/Rab4^Q72L^ when Baf was also provided (Fig. 7B).

### HRES-1/Rab4^Q72L^ promotes the formation of mitochondrial tubular network upon starvation

Mitochondria fuse and form a highly interconnected tubular network upon starvation to resist degradation [24]. Upon starvation of cells transfected with LC3 only, 17±11% of mitochondria assumed tubular shape (Fig. 4A). The formation of highly interconnected tubular mitochondria was enhanced to 49±17% in cells transfected with HRES-1/Rab4^Q72L^ (p = 0.02; Fig. 4E). No other HRES-1/Rab4 isoform increased the formation of mitochondrial tubular network (cumulative data not shown).

## Discussion

**Figure 8:**
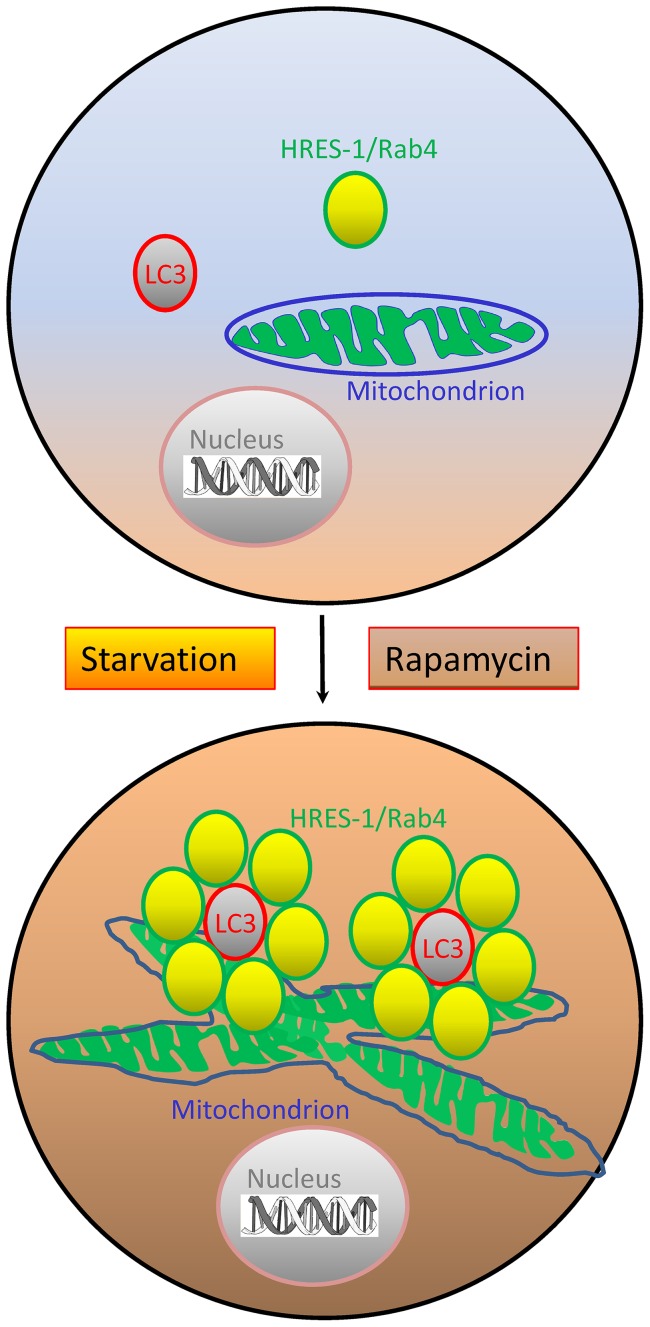
Schematic diagram of the impact by HRES-1/Rab4 on autophagy. HRES-1/Rab4 promotes the formation of LC3^+^ autophagosomes, the accumulation of mitochondria, and their colocalization during autophagy induced by starvation or treatment with rapamycin. LC3^+^ autophagosomes are encircled by HRES-1/Rab4^+^ endosomes. The formation of interconnected mitochondrial tubular networks is enhanced by HRES-1/Rab4^Q72L^ upon starvation.

The present study provides evidence that the endosomal recycling regulator small GTPase HRES-1/Rab4 colocalizes with the autophagosomal membrane component LC3 and mitochondria, and, moreover, it promotes the colocalization between LC3 and mitochondria. These findings are consistent with recent observations that 1) recycling endosomes contribute to the biogenesis of autophagosomal membrane [6], 2) mitochondria and endosomes are juxtaposed under cellular stress [25], and 3) autophagosomes originate from membrane components of mitochondria [3], which may be linked to the turnover of mitochondria, termed mitophagy [26]. Thus, the enhanced colocalization of LC3 and mitochondria by HRES-1/Rab4 may reflect increased formation of autophagosomes. All active isoforms of HRES-1/Rab4, HRES-1/Rab4^1–121^, HRES-1/Rab4^Q72L^ and HRES-1/Rab4^S204Q^, but not the dominant-negative HRES-1/Rab4S27N promoted the accumulation of mitochondria during starvation, suggesting that this small GTPase inhibits mitophagy, particularly under the conditions of cellular stress. Along these lines, blockade of mTOR by Rapa promoted the accumulation of mitochondria in the presence of HRES-1/Rab4^S204Q^, which was reversed by Baf. Since HRES-1/Rab4^S204Q^ cannot be phosphorylated by p34cdc2 kinase and remains endosome-associated throughout the cell cycle [22], this indicates that mTOR blockade may promote the accumulation of mitochondria [16] through interacting with endosome-bound HRES-1/Rab4. This notion is supported by the finding that Rapa-induced colocalization of mitochondria with LC3 was obliterated by HRES-1/Rab4^Q72L^. Rapa also increased mitochondrial mass in the presence of HRES-1/Rab4^Q72L^ when Baf was provided, suggesting that such accumulation of mitochondria may depend on lysosomal function. The marked enhancement of interconnected mitochondrial tubular networks by HRES-1/Rab4^Q72L^ documents its impact on a key checkpoint of mitochondrial preservation during autophagy. Therefore, the colocalization of LC3 with mitochondria upon starvation and the enhancement of this colocalization by HRES-1/Rab4 may represent coordinated steps between autophagosome formation and retention of mitochondria through reduced mitophagy (Fig. 8).

The colocalization of HRES-1/Rab4 with LC3 was enhanced by starvation and Rapa, both of which induce autophagy [14], [15]. The induction of this colocalization was compromised by mutations at S^27^, Q^72^, and S^204^, indicating that this activity requires the complete functional integrity of HRES-1/Rab4. The association of HRES-1/Rab4 with LC3 and mitochondria was also enhanced by Baf. While Baf has been widely used as an inhibitor of autophagy [27], it can also lead to mitochondrial membrane depolarization and mitochondrial damage [28], [29], which is consistent with our findings.

Here, we documented the existence of a novel splice variant of HRES-1/Rab4, encoding a 121 amino-acid-long N-terminal polypeptide, HRES-1/Rab4^1–121^, which exhibited the most robust colocalization with both LC3 and mitochondria relative to all other HRES-1/Rab4 isoforms. The increased localization of HRES-1/Rab4^1–121^ to LC3 and mitochondria was also the least regulated by starvation, Rapa, or Baf, possibly due to the absence of C-terminal prenylation site that allows association with membrane moieties. As HRES-1/Rab4^1–121^ showed the greatest colocalization with LC3 without starvation or Rapa treatment, this truncated isoform may not act through promoting but rather retaining the association of mitochondria with LC3^+^ lysosomes. Alternatively, the strong association of HRES-1/Rab4^1–121^ with LC3 may reflect its targeting for degradation through the autophagy-lysosome pathway [15], [30], which has been also documented for other proteins such as misfolded superoxide dismutase [31] and truncated Cln6 [32].

These findings complement earlier observations about the involvement of early endosomal small GTPases in autophagy [8], such as Rab11 in autophagosome formation [6] and Rab7 in autophagosome progression [10] and maturation [9]. The role of HRES-1/Rab4 in mitophagy may be particularly relevant for the pathogenesis of mitochondrial dysfunction and oxidative stress in T cells of SLE patients [33]. Redox-controlled overexpression of HRES-1/Rab4 in lupus T cells is partially reversed by Rapa [13]. Interestingly, the accumulation of mitochondria in lupus T cells is resistant to mTOR blockade by Rapa [16] or NAC [34]. As shown in this study, the accumulation of mitochondria was induced during starvation by wild-type HRES-1/Rab4, C-terminally truncated HRES-1/Rab4^1–121^, constitutively active/GTPase-deficient HRES-1/Rab4^Q72L^ and phosphorylation-resistant HRES-1/Rab4^S204Q^ but not by dominant-negative/GTP binding-deficient HRES-1/Rab4^S27N^. Thus, the specificity of HRES-1/Rab4-mediated mitochondrial accumulation is indicated through its abrogation by the dominant-negative HRES-1/Rab4^S27N^ mutation. These findings are consistent with recent observations that HRES-1/Rab4 depletes the mitophagy initiator Drp1 and thus facilitates the accumulation of mitochondria [19]. Along these lines, dominant-negative HRES-1/Rab4^S27N^ or pharmacological blockade of HRES-1/Rab4 enhanced Drp1 levels and diminished mitochondrial mass in human and mouse T cells [19]. In accordance with these observations, pharmacological blockade of Drp1 also elicited the accumulation of mitochondria [35].The present study further substantiates the involvement of HRES-1/Rab4 in autophagy, indicating its potential roles in formation of autophagosomes and preservation of mitochondria by reduced mitophagy.

## Materials and Methods

### Vector constructs

HRES-1/Rab4 cDNA was inserted into the multiple cloning site (MCS) of the pAAV-MCS vector fused to the C-terminus of enhanced green fluorescent protein (eGFP; Stratagene, La Jolla, CA) or fluorescent protein 650 (FP650), as described previously [12], [36]. We also made bicistronic constructs having the internal ribosomal entry site positioned between the two open reading frames (pAAV-HRES-1/Rab4-IRES-GFP) [12]. Point mutations were created by site-directed mutagenesis using the PCR-based Quick Change method (Stratagene, La Jolla, CA) to produce wild-type HRES-1/Rab4, GDP-locked HRES-1/Rab4^S27N^ (TCT→AAT), GTP-locked HRES-1/Rab4^Q72L^ (CAA→CTA), HRES-1/Rab4^1–121^ (C-terminally truncated) and cell cycle phosphorylation-defective HRES-1/Rab4^S204Q^ mutants (TCA→CAA). The GDP-locked HRES-1/Rab4^S27N^ prevents GTP binding and creates a dominant-negative mutation [20]. The GTP-locked HRES-1/Rab4^Q72L^ mutant is constitutively active due to elimination of GTPase activity [21]. HRES-1/Rab4^S204Q^ will not be phosphorylated by p34cdc2 kinase in mitotic cells and remains endosome-associated throughout the cell cycle [22]. Amino acid sequence of HRES-1/Rab4^1–121^ represents a 36-nucleotide out-of-frame deletion, which was attributed to alternative splicing (GenBank submission number 1591873). This results in a frameshift with a new protein corresponding to the 96 N-terminal amino acid residues of HRES-1/Rab4 continuing into 25 C-terminal amino acid residues, which are unrelated to the C-terminal 97–218 peptide domain of wild-type HRES-1/Rab4. A schematic diagram of all investigated HRES-1/Rab4 isoforms is shown in Figs. 1A and B.

### Transfections

HeLa cell were cultured in Dulbecco's modified Eagle's medium (DMEM) supplemented with 10% FBS, 2 mM L-glutamine, 100 U/mL penicillin, 100 µg/mL streptomycin, 10 µg/mL amphotericin B. 24 h before transfection. 10^5^ cells per well were seeded in glass bottom dishes Petri dishes (MatTek, Ashland, MA; Cat no. P35GC-1.5-10-C). Cells were washed in PBS and transfected with 1200 ng of DNA in serum-free DMEM for 6 hours using Lipofectamine 2000 reagent and protocol (Life Technologies, Grand Island, NY). After transfection, cells were washed with PBS, incubated in complete DMEM medium for 48 hours, followed by induction of autophagy, and subsequent analyses by microscopy, western blot, and flow cytometry (Fig. S1).

### Autophagy induction

After transfection, cells were washed with PBS and starved with serum-free DMEM and compared to cells maintained in complete DMEM for 4 hours. Parallel cultures were treated for 4 hours with 200 nM bafilomycin A1 (Baf), a lysosomal or vacuolar type H^+^-ATPase inhibitor commonly used to prevent autophagosome-lysosome fusion [37], [38]. However, the effect of Baf on lysosomes may be non-selective, recent studies revealed its mitochondrial toxicity [29], [39]. Before flow cytometry and microscopy, cells were loaded with 100 nM Mito Tracker Deep Red (MTDR) for 30 minutes and washed with PBS twice. Indeed, we observed a 32±5% loss of MTDR fluorescence by flow cytometry after 4 h treatment with Baf (p = 0.001; data not shown). To inhibit mTOR [13], cells were treated by 100 nM rapamycin (Rapa) for 4 hours. Control cultures included 0.1% dimethylsulfoxide (DMSO) used as solvent for Baf and Rapa.

### Flow cytometry

Transfection of expression vectors producing eGFP (excitation: 488 nm, emission: 507 nm; FL1 A channel) and FP650 (excitation: 592 nm, emission: 650 nm; YDC-A channel) and staining of mitochondria with 100 nM MitoTracker Deep Red (MTDR, excitation: 644 nm, emission: 665 nm; Red C-A channel) were monitored by flow cytometry. Samples were analyzed using a Becton Dickinson LSRII flow cytometer equipped with 20 mW solid-state Ng-YAG (emission at 355 nm), 20 mW argon (emission at 488 nm), 10 mW diode-pumped solid-state yellow-green (emission at 535 nm), and 16 mW helium-neon lasers (emission at 634 nm). Data were analyzed with Flow Jo 7.6 software (TreeStar Corporation, Ashland, OR). Dead cells and debris were excluded from the analysis by electronic gating of forward (FSC) and side scatter (SSC) measurements. Each measurement was carried out on ≥10,000 cells.

### Western blot analysis

Cells were trypsinized, washed once with PBS, and resuspended in radio-immunoprecipitation assay buffer (150 mM NaCl, 2% NP-40, 0.5% sodium deoxycholate, 0.1% SDS, 50 mM Tris pH 8.0, 1 mM PMSF, 1 μg/ml aproptinin, 1 μg/ml pepstatin, 1 μg/ml leupeptin, 1 mM NaF, 1 mM sodium orthovanadate, 0.1 mM sodium molybdate, 10 mM sodium pyrophosphate) at a density of 10^7^ cells/ml on ice, followed by addition of equal volumes of Laemmli protein sample buffer (60 mM Tris-Cl pH 6.8, 2% SDS, 10% glycerol, 5% β-mercaptoethanol, 0.01% bromophenol blue) and heated to 95°C for 5 minutes prior to separation on SDS-PAGE gels and transfer to 0.45 μm nitrocellulose membranes. HRES-1/Rab4 was detected by primary rabbit antibodies directed to the C-terminus (Santa Cruz SC312) and Ab 13407 directed to full-length native protein [12]. Expression of HRES-1/Rab4^1–121^ was detected by G1432 rabbit antibody directed to peptide residues 100–121 (Genemed, San Antonio, TX). LC3A/B (#4108), and LC3B (#2775) antibodies were obtained from Cell Signaling. Reactivities to primary antibodies were detected with horseradish peroxidase-conjugated secondary antibodies (Jackson, West Grove, PA) and visualized by enhanced chemiluminescence (Western Lightning Chemiluminescence Reagent Plus, GE Health Care/PerkinElmer Life Sciences, Inc., Boston, Massachusetts). Automated densitometry was used to quantify the relative levels of protein expression using a Kodak Image Station 440CF with Kodak 1D Image Analysis Software (Eastman Kodak Company, Rochester, NY).

### Confocal microscopy

After autophagy induction, culture dishes with adherent cells were kept in PBS on ice until images were taken using a Zeiss LSM 510 confocal microscope and 63x oil objective. Visualization of the specimens on the slides was carried out under a Zeiss 510 LSM Meta confocal microscope using Zeiss LSM Image Browser software version 4.2 (Carl Zeiss Microimaging, Thornwood, NJ). Camera gain was calibrated on cells that have been untransfected and unstained. Sequential scanning was used to record eGFP (excitation: 488 nm, emission: 507 nm), FP650 (excitation: 592 nm, emission: 650 nm), and MTDR (excitation: 644 nm, emission: 665 nm); the RGB images were converted to 8-bit grayscales and pseudo-colored in green, red, and blue, respectively. The means for negative controls were set at 0 intensity. Fluorescence intensities were recorded in a range of 0–255. Fluorescence intensity was determined for each pixel using ImageJ(http://rsbweb.nih.gov/ij/). ImageJ was used to quantify colocalization between eGFP-tagged Rab4, FP650 tagged LC3 and Mitotracker Deep Red loaded mitochondria within cells. Cells were selected and the region of interest (ROI) was defined by an ROI analyzer plugin. Mean intensity values were taken in each channels for each pixel. For each experiment data were generated from analyses of 21.39±0.85 cells (range 6–40 cells). Colocalized signal between Rab4, LC3 and MTDR were quantified and divided by the mean value of the corresponding channel to determine colocalization ratio [40]. Tubular morphology of mitochondria was quantified as earlier described [24].

### Statistical analysis

Data are presented as mean ± SEM, unless stated otherwise. Statistical analyses were done using the Graphpad Prism 5.0 software. Student's t-test was used to analyze the effect of various treatments within a mutation and 2-way ANOVA was done with Bonferroni post-tests to analyze the effect between mutations and treatments. Changes were considered significant at p value<0.05.

## Supporting Information

Figure S1 **Flow cytometry detection of HRES-1/Rab4 isoforms, including wild-type HRES-1/Rab4, C-terminally truncated HRES-1/Rab4^1–121^, dominant-negative/GTP binding-deficient HRES-1/Rab4^S27N^, constitutively active/GTPase-deficient HRES-1/Rab4^Q72L^ and phosphorylation-resistant form HRES-1/Rab4^S204Q^, tagged with eGFP and LC3 fused to FP650 (FP650-LC3).** A) Flow cytometry of HeLa cells transfected with backbone vector pAAV-MCS and expression constructs producing HRES-1/Rab4-eGFP fusion proteins and eGFP alone. B, Flow cytometry of HeLa cells transfected with backbone vector pAAV-MCS, and expression vectors producing FP650-LC3 fusion protein and FP650 alone.
(PDF)

## References

[1] Mizushima N, Levine B, Cuervo AM, Klionsky DJ (2008) Autophagy fights disease through cellular self-digestion. Nature 451: 1069–1075. 18305538 10.1038/nature06639 PMC2670399

[2] Levine B, Mizushima N, Virgin HW (2011) Autophagy in immunity and inflammation. Nature 469: 323–335 10.1038/nature09782. 21248839 10.1038/nature09782 PMC3131688

[3] Hailey DW, Rambold AS, Satpute-Krishnan P, Mitra K, Sougrat R, et al (2010) Mitochondria Supply Membranes for Autophagosome Biogenesis during Starvation. Cell 141: 656–667. 20478256 10.1016/j.cell.2010.04.009 PMC3059894

[4] Metcalf DJ, Garcia-Arencibia M, Hochfeld WE, Rubinsztein DC (2012) Autophagy and misfolded proteins in neurodegeneration. Exp Neurol 238: 22–28. 21095248 10.1016/j.expneurol.2010.11.003 PMC3463804

[5] Axe EL, Walker SA, Manifava M, Chandra P, Roderick HL, et al (2008) Autophagosome formation from membrane compartments enriched in phosphatidylinositol 3-phosphate and dynamically connected to the endoplasmic reticulum. J Cell Biol 182: 685–701. 18725538 10.1083/jcb.200803137 PMC2518708

[6] Longatti A, Lamb CA, Razi M, Yoshimura SI, Barr FA, et al (2012) TBC1D14 regulates autophagosome formation via Rab11- and ULK1-positive recycling endosomes. J Cell Biol 197: 659–675. 22613832 10.1083/jcb.201111079 PMC3365497

[7] Gao W, Kang JH, Liao Y, Ding WX, Gambotto AA, et al (2010) Biochemical isolation and characterization of the tubulovesicular LC3-positive autophagosomal compartment. J Biol Chem 285: 1371–1383. 19910472 10.1074/jbc.M109.054197 PMC2801263

[8] Longatti A, Tooze SA (2012) Recycling endosomes contribute to autophagosome formation. Autophagy 8: 1682–1683. 22874560 10.4161/auto.21486 PMC3494599

[9] Jager S, Bucci C, Tanida I, Ueno T, Kominami E, et al (2004) Role for Rab7 in maturation of late autophagic vacuoles. J Cell Sci 117: 4837–4848. 15340014 10.1242/jcs.01370

[10] Gutierrez MG, Munafo DB, Beron W, Colombo MI (2004) Rab7 is required for the normal progression of the autophagic pathway in mammalian cells. J Cell Sci 117: 2687–2697. 15138286 10.1242/jcs.01114

[11] Mari M, Monzo P, Kaddai V, Keslair F, Gonzalez T, et al (2006) The Rab4 effector Rabip4 plays a role in the endocytotic trafficking of Glut 4 in 3T3-L1 adipocytes. J Cell Sci 119: 1297–1306. 16522682 10.1242/jcs.02850

[12] Nagy G, Ward J, Mosser DD, Koncz A, Gergely P, et al (2006) Regulation of CD4 Expression via Recycling by HRES-1/RAB4 Controls Susceptibility to HIV Infection. J Biol Chem 281: 34574–34591. 16935861 10.1074/jbc.M606301200

[13] Fernandez DR, Telarico T, Bonilla E, Li Q, Banerjee S, Middleton FA, et al (2009) Activation of mTOR controls the loss of TCRζ in lupus T cells through HRES-1/Rab4-regulated lysosomal degradation. J Immunol 182: 2063–2073. 19201859 10.4049/jimmunol.0803600 PMC2676112

[14] Sengupta S, Peterson TR, Sabatini DM (2010) Regulation of the mTOR Complex 1 Pathway by Nutrients, Growth Factors, and Stress. Mol Cell 40: 310–322 10.1016/j.molcel.2010.09.026 20965424 PMC2993060

[15] Tooze SA, Jefferies HBJ, Kalie E, Longatti A, Mcalpine FE, et al (2010) Trafficking and signaling in mammalian autophagy. IUBMB Life 62: 503–508 10.1002/iub.334. 20552641 10.1002/iub.334

[16] Fernandez D, Bonilla E, Mirza N, Perl A (2006) Rapamycin reduces disease activity and normalizes T-cell activation-induced calcium fluxing in patients with systemic lupus erythematosus. Arth Rheum 54: 2983–2988. 16947529 10.1002/art.22085 PMC4034146

[17] Lai Z-W, Borsuk R, Shadakshari A, Yu J, Dawood M, et al (2013) mTOR activation triggers IL-4 production and necrotic death of double-negative T cells in patients with systemic lupus eryhthematosus. J Immunol 191: 2236–2246. 23913957 10.4049/jimmunol.1301005 PMC3777662

[18] Sancak Y, Peterson TR, Shaul YD, Lindquist RA, Thoreen CC, et al (2008) The Rag GTPases Bind Raptor and Mediate Amino Acid Signaling to mTORC1. Science 320: 1496–1501. 18497260 10.1126/science.1157535 PMC2475333

[19] Caza TN, Fernandez D, Talaber G, Oaks Z, Haas M, et al.. (2013) HRES-1/RAB4-Mediated Depletion of DRP1 Impairs Mitochondrial Homeostasis and Represents a Target for Treatment in SLE. Ann Rheum Dis In Press. 10.1136/annrheumdis-2013-203794 PMC4047212 23897774

[20] Lazzarino DA, Blier P, Mellman I (1998) The monomeric guanosine triphosphatase rab4 controls an essential step on the pathway of receptor-mediated antigen processing in B cells. J Exp Med 188: 1769–1774. 9815254 10.1084/jem.188.10.1769 PMC2212406

[21] Cormont M, Meton I, Mari M, Monzo P, Keslair F, et al (2003) CD2AP/CMS Regulates Endosome Morphology and Traffic to the Degradative Pathway Through its Interaction with Rab4 and c-Cbl. Traffic 4: 97–112 10.1034/j.1600-0854.2003.40205.x 12559036

[22] van der Sluis P, Hull M, Webster P, Male P, Goud B, et al (1992) The small GTP-binding protein rab4 controls an early sorting event on the endocytic pathway. Cell 70: 729–740. 1516131 10.1016/0092-8674(92)90307-x

[23] Weidberg H, Shvets E, Elazar Z (2011) Biogenesis and Cargo Selectivity of Autophagosomes. Ann Rev Biochem 80: 125–156 10.1146/annurev-biochem-052709-094552 21548784

[24] Rambold AS, Kostelecky B, Elia N, Lippincott-Schwartz J (2011) Tubular network formation protects mitochondria from autophagosomal degradation during nutrient starvation. Proc Natl Acad Sci USA 108: 10190–10195. 21646527 10.1073/pnas.1107402108 PMC3121813

[25] Calore F, Genisset C, Casellato A, Rossato M, Codolo G, et al (2010) Endosome-mitochondria juxtaposition during apoptosis induced by H. pylori VacA. Cell Death and Differentiation 17: 1707–1716. 20431599 10.1038/cdd.2010.42 PMC3048310

[26] Novak I (2012) Mitophagy: A complex mechanism of mitochondrial removal. Antioxidants and Redox Signaling 17: 794–802. 22077334 10.1089/ars.2011.4407

[27] Alessandri C, Barbati C, Vacirca D, Piscopo P, Confaloni A, et al (2012) T lymphocytes from patients with systemic lupus erythematosus are resistant to induction of autophagy. FASEB J 26: 4722–4732. 22835828 10.1096/fj.12-206060 PMC3475261

[28] Teplova VV, Tonshin AA, Grigoriev PA, Saris NEL, Salkinoja-Salonen MS (2007) Bafilomycin A1 is a potassium ionophore that impairs mitochondrial functions. J Bioenerg Biomembr 39: 321–329. 17917797 10.1007/s10863-007-9095-9

[29] Zhdanov AV, Dmitriev RI, Papkovsky DB (2011) Bafilomycin A1 activates respiration of neuronal cells via uncoupling associated with flickering depolarization of mitochondria. Cell Mol Life Sci 68: 903–917. 20820851 10.1007/s00018-010-0502-8 PMC3037485

[30] Klionsky DJ, Baehrecke EH, Brumell JH, Chu CT, Codogno P, et al (2011) A comprehensive glossary of autophagy-related molecules and processes (2 nd edition). Autophagy 7: 1273–1294. 21997368 10.4161/auto.7.11.17661 PMC3359482

[31] Hadano S, Otomo A, Kunita R, Suzuki-Utsunomiya K, Akatsuka A, et al.. (2010) Loss of ALS2/Alsin exacerbates motor dysfunction in a SOD1H46R-expressing mouse ALS model by disturbing endolysosomal trafficking. PLoS ONE 5. 10.1371/journal.pone.0009805 PMC2842444 20339559

[32] Thelen M, Dae M, Schweizer M, Hagel C, Wong AMS, et al.. (2012) Disruption of the autophagy-lysosome pathway is involved in neuropathology of the nclf mouse model of neuronal ceroid lipofuscinosis. PLoS ONE 7. 10.1371/journal.pone.0035493 PMC3335005 22536393

[33] Perl A (2013) Oxidative stress in the pathology and treatment of systemic lupus erythematosus. Nat Rev Rheumatol 9: 674–686. 24100461 10.1038/nrrheum.2013.147 PMC4046645

[34] Lai Z-W, Hanczko R, Bonilla E, Caza TN, Clair B, et al (2012) N-acetylcysteine reduces disease activity by blocking mTOR in T cells of lupus patients. Arth Rheum 64: 2937–2946. 22549432 10.1002/art.34502 PMC3411859

[35] Kim B, Kim JS, Yoon Y, Santiago MC, Brown MD, et al (2013) Inhibition of Drp1-dependent mitochondrial division impairs myogenic differentiation. Am J Physiol Regul Integr Comp Physiol 305: R927–R938. 23904108 10.1152/ajpregu.00502.2012

[36] Shcherbo D, Shemiakina II, Ryabova AV, Luker KE, Schmidt BT, et al (2010) Near-infrared fluorescent proteins. Nat Meth 7: 827–829 10.1038/nmeth.1501. 10.1038/nmeth.1501 PMC4425247 20818379

[37] Marceau F, Bawolak MT, Lodge R, Bouthillier J, Gagne-Henley A, et al (2012) Cation trapping by cellular acidic compartments: Beyond the concept of lysosomotropic drugs. Toxicol Appl Pharmacol 259: 1–12. 22198553 10.1016/j.taap.2011.12.004

[38] Klionsky DJ, Elazar Z, Seglen PO, Rubinsztein DC (2008) Does bafilomycin A1 block the fusion of autophagosomes with lysosomes? Autophagy 4: 849–850. 18758232 10.4161/auto.6845

[39] Zhdanov AV, Dmitriev RI, Papkovsky DB (2012) Bafilomycin A1 activates HIF-dependent signalling in human colon cancer cells via mitochondrial uncoupling. Biosci Rep 32: 587–595. 22943412 10.1042/BSR20120085 PMC3497721

[40] Mancuso MR, Davis R, Norberg SM, O'Brien S, Sennino B, et al (2006) Rapid vascular regrowth in tumors after reversal of VEGF inhibition. J Clin Invest 116: 2610–2621. 17016557 10.1172/JCI24612 PMC1578604

