# Supplementary material for: HRES-1/Rab4 Promotes the Formation of LC3+ Autophagosomes and the Accumulation of Mitochondria during Autophagy
Source: PLoS One. 2014 Jan 3;9(1):e84392. doi: 10.1371/journal.pone.0084392 (PMC3880286; doi:10.1371/journal.pone.0084392)
Supplement: Figure S1 — Flow cytometry detection of HRES-1/Rab4 isoforms, including wild-type HRES-1/Rab4, C-terminally truncated HRES-1/Rab41–121, dominant-negative/GTP binding-deficient HRES-1/Rab4S27N, constitutively active/GTPase-deficient HRES-1/Rab4Q72L and phosphorylation-resistant form HRES-1/Rab4S204Q, tagged with eGFP and LC3 fused to FP650 (FP650-LC3). A) Flow cytometry of HeLa cells transfected with backbone vector pAAV-MCS and expression constructs producing HRES-1/Rab4-eGFP fusion proteins and eGFP alone. B, Flow cytometry of HeLa cells transfected with backbone vector pAAV-MCS, and expression vectors producing FP650-LC3 fusion protein and FP650 alone. (PDF) [file pone.0084392.s001.pdf]

LEGEND TO SUPPLEMENTARY FIGURE S1

Fig. S1. Flow cytometry detection of HRES-1/Rab4 isoforms, including wild-type HRES-1/Rab4, C-terminally truncated HRES-1/Rab4$^{1-121}$, dominant-negative/GTP binding-deficient HRES-1/Rab4^{S27N}, constitutively active/GTPase-deficient HRES-1/Rab4^{Q72L} and phosphorylation-resistant form HRES-1/Rab4^{S204Q}, tagged with eGFP and LC3 fused to FP650 (FP650-LC3). A) Flow cytometry of HeLa cells transfected with backbone vector pAAV-MCS and expression constructs producing HRES-1/Rab4-eGFP fusion proteins and eGFP alone. B, Flow cytometry of HeLa cells transfected with backbone vector pAAV-MCS, and expression vectors producing FP650-LC3 fusion protein and FP650 alone.

Figure S1

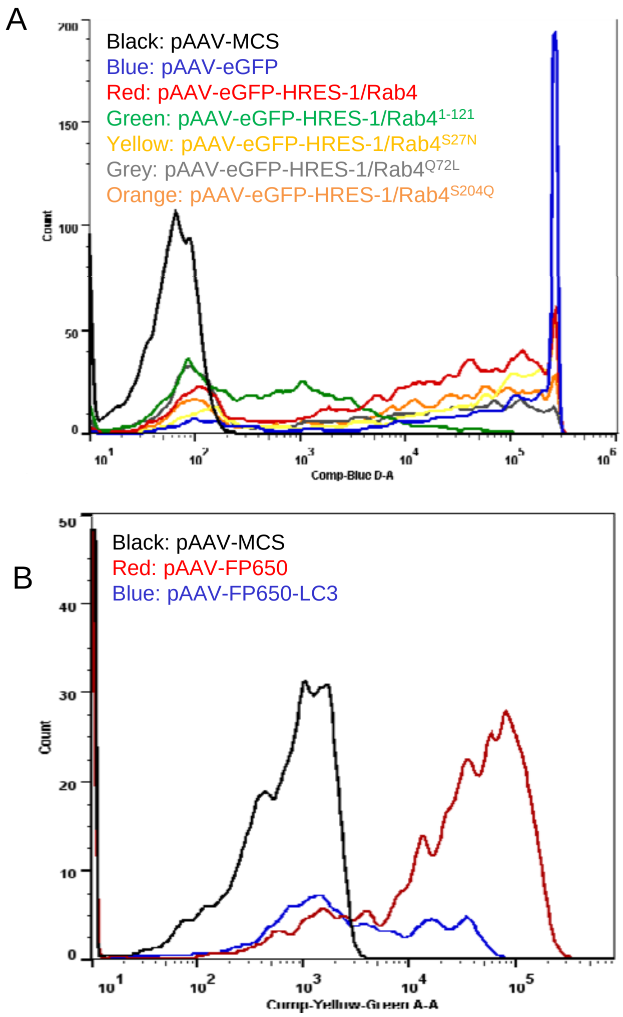
A
Black: pAAV-MCS
Blue: pAAV-eGFP
Red: pAAV-eGFP-HRES-1/Rab4
Green: pAAV-eGFP-HRES-1/Rab4$^{1-121}$
Yellow: pAAV-eGFP-HRES-1/Rab4^{S27N}
Grey: pAAV-eGFP-HRES-1/Rab4^{Q72L}
Orange: pAAV-eGFP-HRES-1/Rab4^{S204Q}
Count
200
150
100
50
0
10^1 10^2 10^3 10^4 10^5 10^6
Comp-Blue D-A
B
Black: pAAV-MCS
Red: pAAV-FP650
Blue: pAAV-FP650-LC3
Count
50
40
30
20
10
0
10^1 10^2 10^3 10^4 10^5
Comp-Yellow-Green A-A
